# CFD of roughness effects on laminar heat transfer applied to additive manufactured minichannels

**DOI:** 10.1007/s00231-022-03268-1

**Published:** 2022-08-04

**Authors:** Mohammadreza Kadivar, David Tormey, Gerard McGranaghan

**Affiliations:** 1 Centre for Precision Engineering Material and Manufacturing Research (PEM Centre), Atlantic Technological University, Sligo, F91 YW50 Ireland; 2 I-Form, The SFI Advanced Manufacturing Research Centre, Dublin, Ireland

## Abstract

Additive manufacturing has received significant interest in the fabrication of functional channels for heat transfer; however, the inherent rough surface finish of the additively manufactured channels can influence thermal performance. This study investigates the impact of roughness on the thermo-fluid characteristics of laminar forced convection in rough minichannels. A numerical model was developed to create 3D Gaussian roughness with specified root-mean-square height. The finite volume method was used to solve the conjugate heat transfer of developed laminar flow in square minichannels. For Reynolds numbers ranging from 200 to 1600, the simulation results indicated enhanced heat transfer and increased flow resistance as Reynolds number increases, compared to a smooth minichannel, where effects on heat transfer and flow friction were negligible. For channels with relative roughness (root-mean-square height to channel hydraulic diameter) of 0.0068, 0.0113, and 0.0167, increasing the Reynolds number led to increased friction factor by 1.56, 1.71, and 2.91%, while the Nusselt number was enhanced up to 0.03%, 32.74%, and 46.05%, respectively. Heat transfer reduced in roughness valleys due to the presence of local low-velocity fluid in these regions; however, recirculation regions can occur in deep valleys of high roughness, increasing heat transfer and flow friction. Heat transfer was enhanced over roughness peaks due to flow impingement on the windward face of roughness as well as intensified energy transfer to the channel wall from roughness. Moreover, surfaces with higher roughness have a greater number of high peaks providing a thermal-flow path of a larger area and a thermal conductivity greater than that of the fluid.

## Introduction

Rapid technological advancements have spurred a requirement for high-performance and compact thermal management systems [1]. In this regard, micro-and mini-channel heat exchangers have become popular owing to their high energy efficiency [2], low mass, small volume, and high heat removal capacity [3]. Micro- and mini-channel heat exchangers have gained widespread application in refrigeration [4], air-conditioning [4], electronic cooling [5], turbine blade cooling [6], conformal cooling [7], and energy conversion [8]. Based on the Kandlikar and Grande [9] classification, microchannels have hydraulic diameters ranging from 10 to 200 μm, while those of minichannels range from 200 μm to 3 mm. A great deal of research devoted to fluid flow and heat transfer in mini/microchannels that can be found in review papers in Refs. [10–12].

Traditional methods of fabrication of small mini/microchannels include lithography, wet bulk micromachining and surface micromachining [13]; however, such methods experience limitations during the fabrication of complex channels for applications such as conformal cooling. Additive Manufacturing (AM), particularly Powder Bed Fusion (PBF), have provided the capacity to fabricate metallic devices with a high degree of complexity [14] in a single-step manufacturing operation [15]. PBF technologies permit superior flexibility to fabricate complex functional channels for thermal management systems [7, 16], including mini- and micro-channels [17–19]. Many researchers have employed this capacity to create cooling channels with enhanced thermal performance features such as waviness [20], interconnectivity [21], permeability [22], internal pin fins [23], and longitudinal vortex generators [24].

As a natural consequence of metal PBF processes, the surface finish of the fabricated channels is of an irregular roughness [25] which can play a crucial role in transport phenomena, including heat and momentum transfer in laminar, laminar-turbulent transition, and turbulent flows [26, 27]. For laminar internal flows, the surface roughness effects depend on the ratio of roughness height to the characteristic length of the system, known as relative roughness. Reducing the channel’s characteristic length (e.g. hydraulic diameter) can influence the impact of the surface roughness on the fluid flow and heat transfer [28, 29]. Stimpson et al. [18] additively manufactured ten different minichannels using PBF technology and reported that high relative roughness led to increased heat transfer and pressure drop in both laminar and turbulent flows, although their studies mainly focused on turbulent flow.

Roughness-induced flow resistance in laminar flows has been investigated by many researchers [11]. Mohiuddin Mala et al. [30] experimentally studied water flow through rough microtubes in diameters from 50 to 254 μm, finding that, for *Re* < 1000, the pressure drop approximated values predicted by conventional Poiseuille flow theory. However, For *Re* > 1000, they observed a significant departure in flow characteristics from conventional theory predictions. Pfund et al. [31] also measured a friction factor value significantly higher than the classical value for smooth channels. Li et al. [32] conducted an experimental and numerical study of channels with relative roughness of 2.4%, 1.4%, and 0.95% at Reynolds numbers ranging from 20 to 2400. They observed increased pressure drop for relative roughness larger than 1.4%. A summary of studies on the increased flow friction in rough microchannels can be found in Ref. [33]. Dai et al. [11], in their review on liquid flow in rough micro-and minichannels, suggested that, when the relative roughness is less than 1%, roughness has no apparent effect on the friction factor and the critical Reynolds number. They also reported that the channel cross-section has only a marginal impact on the same.

Experimental studies on the heat transfer in rough micro/mini channels with trapezoidal [34], rectangular [35] and circular [36] sections have demonstrated that roughness can enhance heat transfer. Experimental records of heat transfer and pressure drop are influenced by many factors, such as the entrance effect, roughness effect, and uncertainties and errors in instrumentation [37]. Even very accurate measurements may often be overshadowed by geometry measurement uncertainties [38]. A review of the experimental studies can be found in Morini [10].

The effect of surface roughness has also been investigated numerically [32, 33, 39–60], with various methods. Several studies have proposed roughness models to account for roughness in laminar flows. Qu et al. [61] proposed a roughness-viscosity model to account for the difference between the Nusselt number obtained from experiments and predicted by numerical simulation. Koo et al. [62, 63] suggested a concept of an equivalent porous medium layer (PML) by replacing the roughness with a porous film. Based on the concept of the PLM model and discrete element approach of Taylor et al. [64]. Gamrat et al. [68] proposed a Rough Layer Model (RLM) for fully developed laminar flow in microchannels.

For an accurate prediction of fluid dynamics and heat transfer, the geometry of the actual roughness must be resolved in the computation [65], the most important step of which is to generate the roughness. Several studies have used simple abstraction using various regular geometry forms [46, 49, 54–56, 66–68]. However, using regular elements to represent roughness has several issues, the most important being the impact of the shape and arrangement of the roughness elements on the results [54, 55, 66]. Croce and D'Agaro [49] reported that numerical results can be improved by using random-sized rectangular and triangular elements. Another issue is the diversity of the heat transfer enhancement using various elements [55, 66, 68].

The actual geometry of most of the engineering roughness, especially in AM, is random and irregular, a crucial characteristic that should be accounted for in roughness studies. Several methods have been used in the literature to generate 2D and 3D random roughness, including Fourier transform [48], Gaussian function [33, 60, 69], fractal function [39, 40, 43], and random displacement [41]. 2D roughness fails to replicate the actual roughness [33, 70]; therefore, the investigation of the roughness effect on fluid flow and heat transfer requires a 3D model of random roughness, the Gaussian function being the most used approach. Pelević and van der Meer [33] conducted a numerical study of laminar flow in microchannels using the Gaussian function to generate 3D roughness. They showed a maximum of 7% increase in Poiseuille number for relative roughness of 2.93% compared to the smooth channel and a corresponding 4% heat transfer enhancement. Xiong et al. [69] numerically studied heat transfer in a microtube using roughness geometry with Gaussian distribution and observed negligible heat transfer enhancement from roughness. Lu et al. [42] also used the Gaussian function to generate random 3D roughness in a microchannel at *Re* = 400. They observed that the pressure drop increases monotonically and the average Nusselt number remains unchanged with increasing relative roughness up to 1.2%, after which the Nusselt number increases with roughness. These studies suggest that depending on the relative roughness and Reynolds number, roughness can be detrimental or beneficial to the thermal performance of microchannels. Kadivar et al. [71] showed that due to roughness structure, the local heat transfer coefficient on the rough surface fluctuates around an average value, which can vary depending on the Reynolds number. Their study showed that this average value can be higher than the average heat transfer coefficient of a smooth channel.

Most of the previous studies have focused on the effect of roughness in microchannel heat exchangers while the roughness effect on heat transfer in minichannels has received less attention. Moreover, the mechanisms that influence fluid dynamics and heat transfer have not been fully understood. This present paper is an extension of our previous study [71] and investigates the Computational Fluid Dynamics (CFD) of fully developed laminar convective heat transfer in a rough minichannel. A general numerical method for creating random irregular roughness based on the roughness profile was developed and the Gaussian function was utilised for the roughness profile. The impact of surface roughness of several heights on the characteristics of heat transfer and fluid flow were investigated at various Reynolds numbers. The thermal performance of the rough minichannels was evaluated to elucidate roughness effects debated in the literature and ascertain whether roughness is beneficial or deleterious to convective laminar heat transfer. Finally, flow visualisation by CFD was used to reveal the heat transfer and fluid flow phenomena within rough minichannels.

## Geometry

The procedure mentioned in Appendix A with a Gaussian Autocorrelation Function (ACF) was used to generate random heightmaps with various root-mean-square height, ${S}_{q}$.

Depending on the design parameters of AM machine, roughness heights ranging from 5 $\mu m$ to almost 40 $\mu m$ are expected (see Ref. [72]); for instance, Stimpson et al. [18] built AM minichannels with ${S}_{q}$ ranging from 12.6 $\mu m$ to 16.69 $\mu m$. In the present study, different surfaces with roughness heights ranging from ${S}_{q}$ = 8.9 $\mu m$ to 21.0 $\mu m$ (common roughness for AM minichannels) were utilised. A numerical code was developed in MATLAB to generate the heightmap, surface mesh, and STL (Standard Tessellation Language) file. The rough minichannels were created using the method explained in Ref. [71], as follows.

1. generate a heightmap, representing the distribution of roughness heights;
2. create a surface mesh from the heightmap, representing the rough surface;
3. export the surface mesh to an STL file (with a triangular surface mesh); and
4. import the STL file in CAD software to create the channels.

**Fig. 1:**
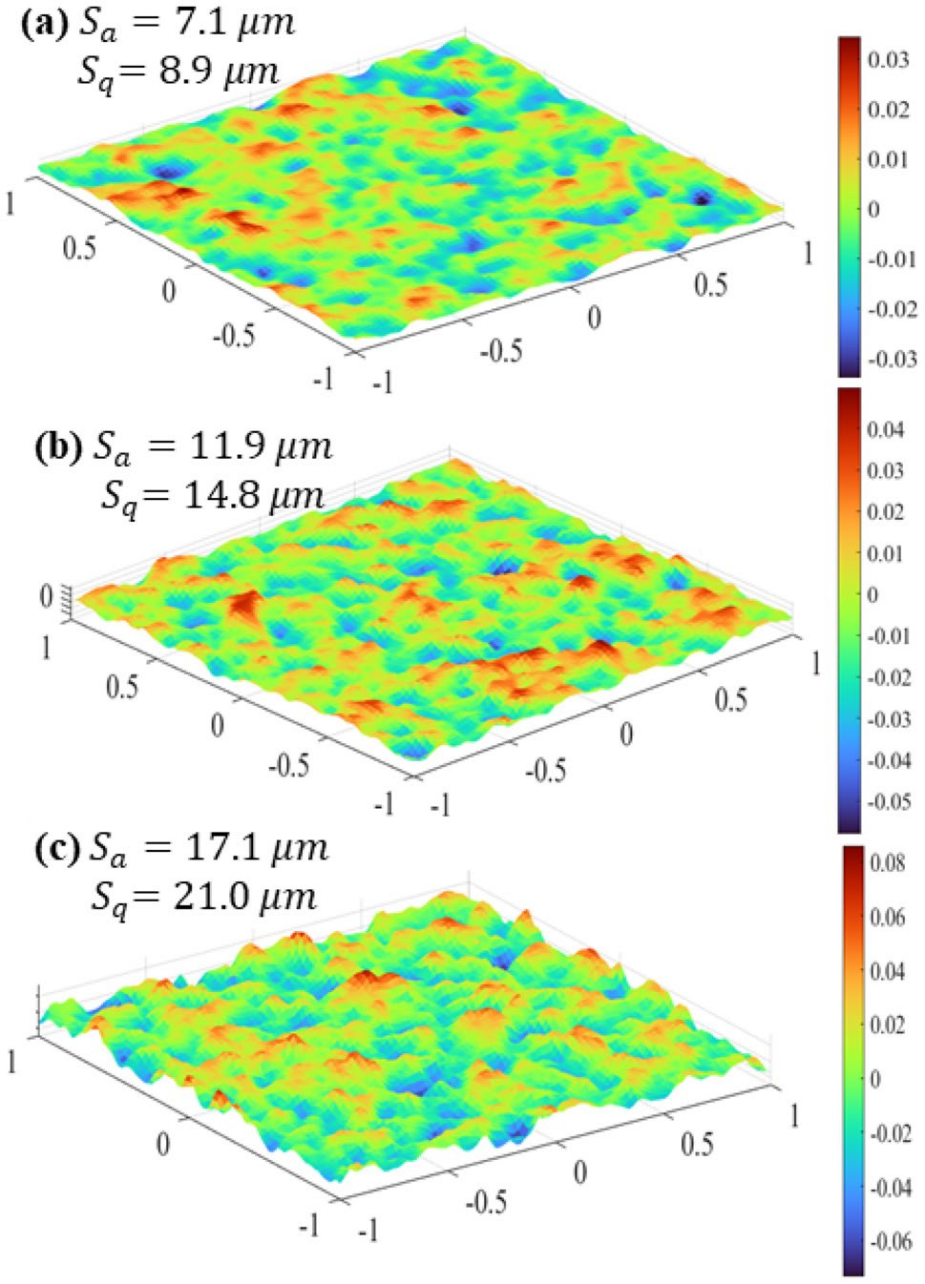
Rough mesh surfaces generated in MATLAB used in (**a**) RMC1, (**b**) RMC2, (**c**) and RMC3, as characterised in Table 1

Figure 1 illustrates four rough mesh surfaces generated with an Autocorrelation Length (ACL) of 50 $\mu m$ in MATLAB. The geometry of the rough minichannels was created in ANSYS SpaceClaim CAD Software, as listed in Table 1 as Rough MiniChannels 1 to 3 (RMC1 to RMC3). ${S}_{q}/{D}_{h}$ was selected as the parameter representing the relative roughness in the channels. Due to the roughness structure, the hydraulic diameter of the rough minichannels reduces, and the heat transfer area from a rough surface $({A}_{R})$ is greater than that of the smooth surface $({A}_{S})$, as shown in Table 1. A smooth minichannel (SMC0) was simulated as a baseline study.

**Table 1:** Specification of rough minichannels used in this study

Minichannel	${S}_{a}(\mu m)$	${S}_{q}(\mu m)$	${D}_{h}$(mm)	${S}_{a}/{D}_{h}$	${S}_{q}/{D}_{h}$	${A}_{R}/{A}_{S}$(%)
SMC0	0.0	0.0	1.3333	0.0	0.0	1.00
RMC1	7.1	8.9	1.3203	0.0054	0.0068	1.21
RMC2	11.9	14.8	1.3091	0.0091	0.0113	7.05
RMC3	17.1	21.0	1.2713	0.0134	0.0167	17.01

**Fig. 2:**
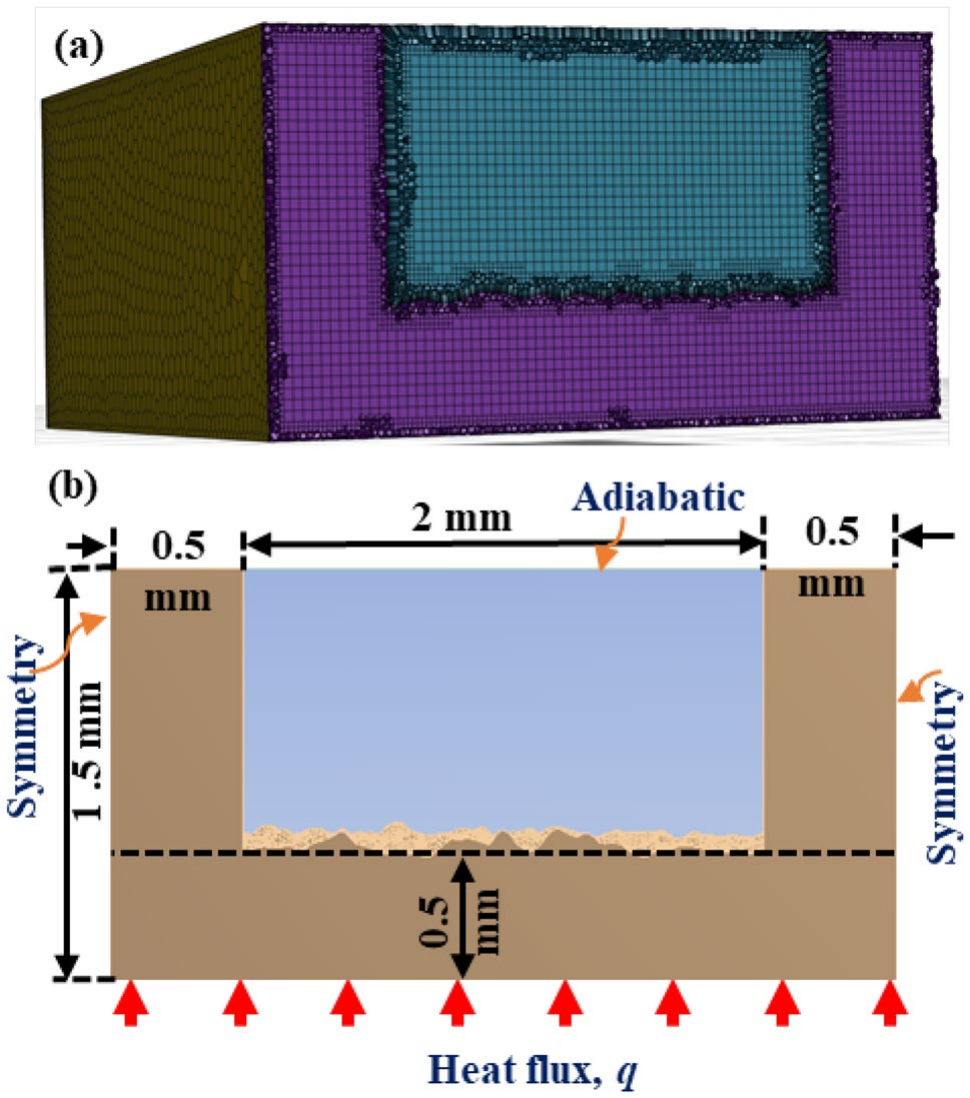
**a** Geometry and mesh; **b** dimensions and boundary conditions

Figure 2(a) shows the geometry and the computational mesh used in this study with dimensions (a height of 1 mm, width of 2 mm, length of 8 mm, and thickness of 0.5 mm) and boundary conditions of the minichannels in Fig. 2(b). The bottom surface of the channel was rough, and the other surfaces were smooth. Generating high-quality mesh on irregular geometries, such as roughness, is challenging. In the present study, the mosaic meshing strategy was used to generate a high-quality mesh in minichannels by generating prism boundary layer cells over surfaces (to capture fluid physics in the wall boundary layer), structured hexagonal elements in the core, and unstructured polyhedral transitional cells between the boundary layer and core mesh. Using mosaic meshing can lead to a 46% reduction in mesh size, a 47.09% increase in computational speed in Fluent and a substantial improvement in mesh quality [73].

## Mathematical model

This section presents the CFD method, including the mathematical representation of governing equations with the boundary conditions, and the method for calculating the flow and heat transfer characteristics. The numerical procedure, mech dependency study, and CFD code validation are also discussed.

### Governing equations

The fluid flow and heat transfer were simulated by solving the Navier–Stokes and energy equations for laminar Newtonian incompressible flow with constant thermophysical properties. The governing equations for convective heat transfer can be expressed as

1 $$\overrightarrow{\nabla }.\overrightarrow{V}=0$$

2 $$\rho \overrightarrow{V}.\left(\overrightarrow{\nabla }\overrightarrow{V}\right)=-\overrightarrow{\nabla }p+\mu \overrightarrow{\nabla }.\left(\overrightarrow{\nabla }\overrightarrow{V}\right)$$

3 $$\rho {c}_{p}\overrightarrow{V}.\left(\overrightarrow{\nabla }T\right)=k{\overrightarrow{\nabla }}^{2}T$$

where $\rho$, $\mu$, ${c}_{\mathrm{p}}$, and $k$ are the density, viscosity, specific heat capacity, and thermal conductivity, while $\overrightarrow{V}$, $p$, and $T$ represent the velocity vector, pressure, and temperature.

The heat transfer in solid can be described the Laplace's equation, expressed as

4 $${\overrightarrow{\nabla }}^{2}T=0$$

### Boundary conditions

Periodic flow boundary conditions were applied to the channel’s inlet and outlet. The flow is driven in the simulation by prescribing a Reynolds number at the inlet and hence prescribing the flowrate as $\dot{m}=Re{A}_{c}\mu /{D}_{h}$, where ${A}_{\mathrm{c}}$ is the channel’s cross-sectional area. The hydraulic diameter, ${D}_{h}$, is defined as

5 $${D}_{h}=\frac{4{A}_{\mathrm{c}}}{{P}_{\mathrm{c}}}$$

where, ${P}_{\mathrm{c}}$ is the circumference of the channel. An average temperature of 300 K was used for the periodic boundary condition for a range of Reynolds numbers from 200 to 1600. A heat flux of 10 W/cm^2^ was applied to the bottom of the minichannels, as shown in Fig. 2. The top wall was assumed to be adiabatic and symmetry boundary condition was applied to the other external walls. The no-slip condition was imposed on the internal walls in contact with the fluid. The conjugate heat transfer approach was employed on the fluid–solid interface, using the interface condition as

6 $${k}_{s}\frac{\partial T}{\partial n}={q}_{f}$$

where $\partial T/\partial n$ is the temperature gradient normal to the wall, ${k}_{s}$ is the thermal conductivity of the solid wall, and ${q}_{f}$ is the heat flux given to the fluid due to the convection. The material of the channel body was AlSi10Mg (${k}_{s}=160 \mathrm{W}/\mathrm{m}.\mathrm{K}$), a common material in the fabrication of heat exchangers by AM. The working fluid is water with thermophysical properties of $\rho =996.6$ kg/m^3^, $\mu =0.00086$ kg/m.s, ${c}_{\mathrm{p}}=4178.5$ J/kg.K, and $k=0.613$ W/m.K, and $Pr=5.86$.

### Data reduction

The heat transfer enhancement of the different channels was determined in terms of the average Nusselt number with respect to the base area (${Nu}_{b}$) and that with respect to the total wetted surface area (${Nu}_{t}$).

7 $${Nu}_{b}=\frac{Q{D}_{h}}{{A}_{b}k({T}_{w}-{T}_{\mathrm{f}})}$$

8 $${Nu}_{t}=\frac{Q{D}_{h}}{{A}_{t}k({T}_{w}-{T}_{\mathrm{f}})}$$

where $Q$ is the total heating power, while ${A}_{b}$ is the base surface (the surface area of the smooth channel), and ${A}_{t}$ total wetted surface area (the area of fluid–solid interface). ${T}_{w}$ is the area-averaged temperature of fluid–solid interface and ${T}_{\mathrm{f}}$ is the volume-averaged fluid temperature, calculated by

9 $${T}_{w}=\frac{{\iint }_{\mathrm{wall}}TdA}{{\iint }_{\mathrm{wall}}dA}$$

10 $${T}_{\mathrm{f}}=\frac{{\iiint }_{\mathrm{fluid}}Td\forall }{{\iiint }_{\mathrm{fluid}}d\forall }$$

where $\forall$ is the fluid volume.

The local heat transfer coefficient is defined as

11 $$h=\frac{q}{(T-{T}_{in})}$$

where *q* is local heat flux, $T$ is local surface temperature, and ${T}_{in}=300K$ is the average inlet temperature.

The Darcy friction factor (*f*) is calculated by

12 $$f=\frac{2{D}_{\mathrm{h}}\Delta p }{\rho L{U}^{2}}$$

where $\Delta p$ is the pressure drop, $L$ is the length of the channel, and $U$ is the average velocity of the fluid. The form drag coefficient $({C}_{D})$ and viscous coefficient $({C}_{\nu })$ over roughness are calculated by

13 $${C}_{D}=\frac{2{F}_{D} }{\rho {A}_{s}{U}^{2}}$$

14 $${C}_{\nu }=\frac{2{F}_{\nu } }{\rho {A}_{s}{U}^{2}}$$

where ${A}_{s}$ is the roughness area, while ${F}_{D}$ and ${F}_{\nu }$ are form and viscous drag forces, respectively.

The performance of rough minichannels, in terms of the combined heat transfer enhancement and pressure loss, can be evaluated by the thermal performance factor (PF), defined as

15 $$\mathrm{PF}=\frac{{Nu}_{t}/{Nu}_{0}}{{\left(f/{f}_{0}\right)}^{(1/3)}}$$

where the subscript *0* refers to the smooth minichannel.

### Numerical procedure and validation

The Finite Volume Method (FVM) with the pressure–velocity coupled algorithm was employed in ANSYS fluent 19.1 to solve the governing equations. The second-order upwind method was used to discretise the transport equations. The convergence of the numerical solution was assured by monitoring the normalised residual of all variables to reach a constant level below 10^−6^.

#### Mesh dependency study

A mesh dependency study was performed to ensure that the CFD model is independent of the mesh size, and followed a series of simulations with subsequent grid refinement, as shown in Table 2 until further mesh refinement does not lead to a significant change in CFD results. A computational grid with 971,254 cells was selected for the rough minichannels used in the present study since further grid refinement leads to only 0.002% and only 0.03% changes in the Nusselt number and friction factor, respectively, which does not justify the increase in computational effort. The similar procedure was repeated to obtain the required mesh for other channels.

**Table 2:** Mesh refinement process for RMC3

Grid	Prism layers	Element size ($\mu m)$		Number of cells	${Nu}_{t}$	$f$	Error in Nu (%)	Error in *f* (%)
		Fluid	Solid					
1	2	80	80	552628	3.913	0.61593	-	-
2	5	50	50	971254	4.128	0.61408	5.49	0.30
3	6	30	40	1270594	4.127	0.61393	0.002	0.02

#### Validation

**Fig. 3:**
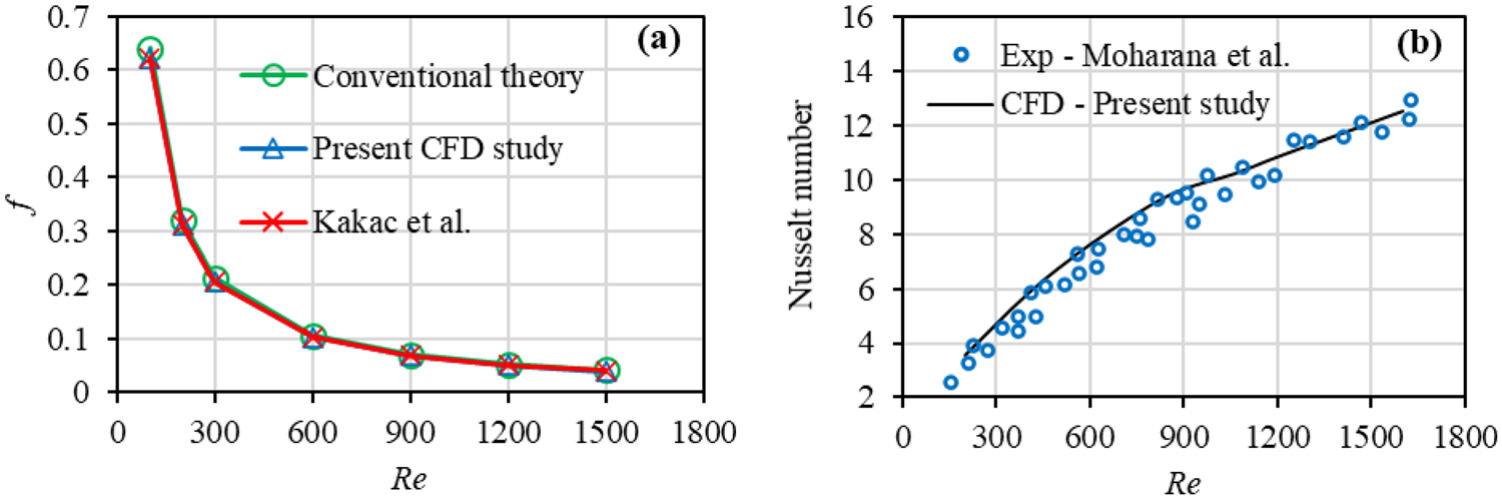
Code validation; **a** comparison of the friction factor between CFD of the present study and Kakac et al. [74]; **b** comparison of average Nusselt number between CFD of the present study and experimental data of Moharana et al. [75]

The CFD method was validated against the experimental data and conventional theory for the smooth channel (SMC0). Figure 3(a) compares the friction factor between CFD of the present study with the conventional theory ($f=64/Re$). A Normalized Root Mean Square Error (NRMSE) of 4.27% was observed between the friction factor of CFD and conventional theory. Since the channel’s aspect ratio influences the friction factor in laminar flows, the results of the friction factor are also compared with the correlation of Kakac et al. [74] ($f=62/Re$) for laminar flow in channels with the aspect ratio of 2 (similar to current study). An excellent agreement with the data of Kakac et al. [74] with an NRMSE of 0.55% was obtained. Figure 3(b) shows a good agreement between the average Nusselt number of CFD and experimental data of Moharana et al. [75] with an NRMSE of 5.49%.

## Results and discussion

The choice of effective length scale is a widely debated issue in literature and has a strong effect on global parameters. The channel’s hydraulic diameter reduces by increasing the roughness height (see Table 1), which is crucial and requires inclusion in the calculations. Therefore, in the present study, the Reynolds number was calculated based on the hydraulic diameter of the channels, calculated as

16 $$Re=\frac{\rho u{D}_{\mathrm{h}} }{\mu }$$

where $\rho$, $\mu$ are fluid density and viscosity, respectively, ${D}_{\mathrm{h}}$ is the channel’s hydraulic diameter, and $u$ is the fluid average velocity.

According to Dai et al. [11], the critical Reynolds number, ${Re}_{c}$, in rough channels can be calculated by

17 $${Re}_{c}=\begin{cases}2300 &0<\varepsilon /{D}_{h}<0.01 \\ 2300-12000(\frac{\varepsilon }{{D}_{h}}-0.001) &0.001<\varepsilon /{D}_{h}<0.2\end{cases}$$

where $\varepsilon /{D}_{h}$ is the relative roughness which is defined as ${S}_{q}/{D}_{h}$ in the present study. By considering the values in Table 1, the critical Reynold numbers for SMC0, RMC1, RMC2, and RMC3 were calculated as 2300, 2230.4, 2176.4, and 2111.6, respectively. Since the maximum Reynolds number considered in the present study (1600) is adequately below the reported critical Reynolds numbers, no turbulence effects are generated that could lead to flow unsteadiness.

**Fig. 4:**
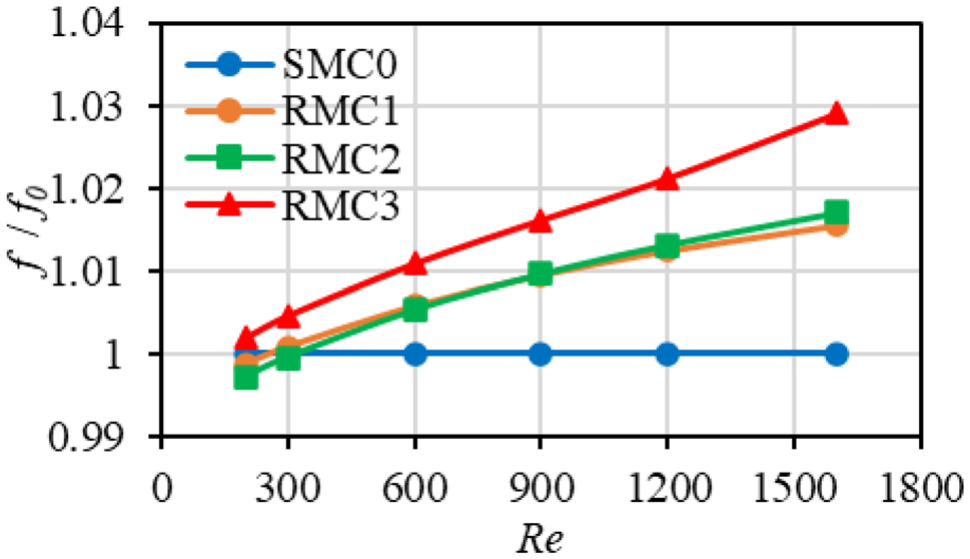
Variation of friction factor ratio with Reynolds number

Figure 4 illustrates the variation of the friction factor ratio with the Reynolds number ranging from 200 to 1600. The vertical axis represents the friction factor ratio of the rough channels ($f$) and baseline smooth channel (${f}_{0}$). The friction factor of the rough channels increases with Reynolds number, which was also observed in experiments of Stimpson [18] and numerical results of Pelević and van der Meer [33]. At *Re* = 200, the friction factor values of RMC1 is smaller than that of the smooth channel (SMC0), and that of RMC2 is even smaller than the RMC1 value. The reason may be due to the existence of low-velocity fluid without recirculation that occurs in roughness valleys and in the spaces between roughness elements which reduces the viscous flow friction (see Fig. 6) and heat transfer. Figure 4 also demonstrates that the flow friction increases with the roughness height; however, the friction factor values are of low magnitude, indicating the absence of complex flow patterns in the channels (see Ref. [76]). Compared to SMC0, at *Re* = 1600 the friction factors of RMC1, RMC2, and RMC3 increases by about 1.56, 1.71, and 2.91%, respectively. The friction factor increases in RMC1 and RMC2 is marginal indicating a negligible or total absence of recirculation regions behind the roughness elements. However, the presence of recirculation regions in RMC3, although of low magnitude, is expected due to the higher friction factor witnessed.

**Fig. 5:**
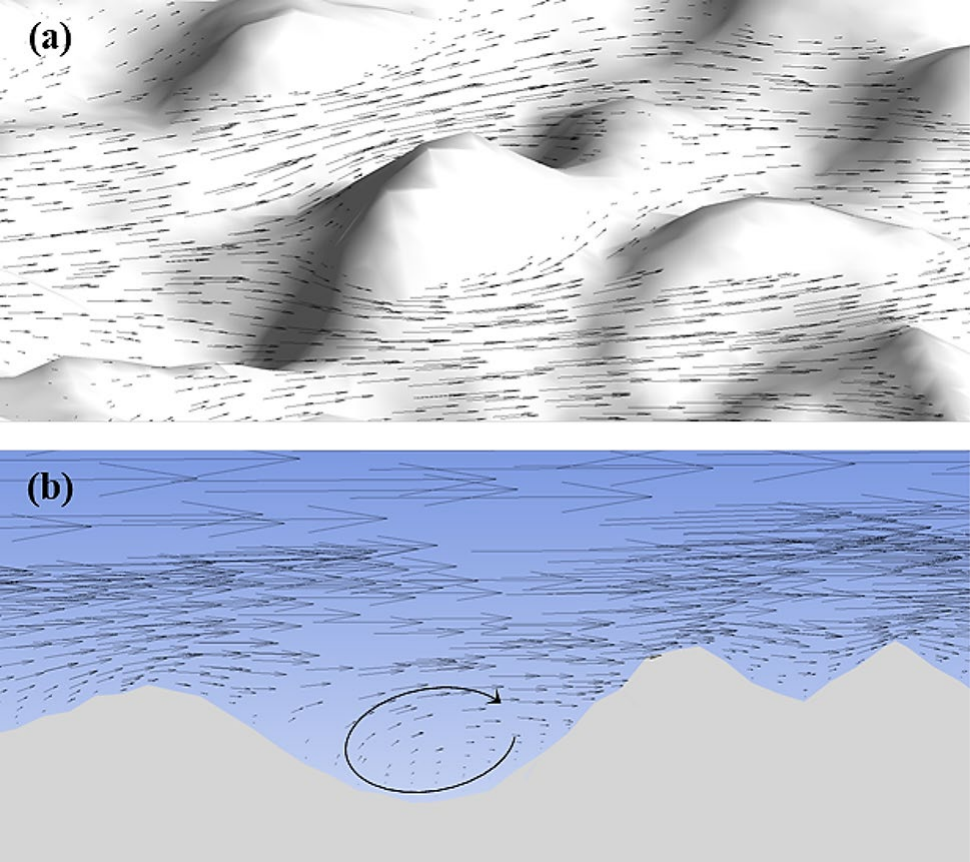
Segment of the velocity vector field **a** around roughness elements, and **b** on a 2D plane normal to the rough surface in RMC3

Figure 5(a) shows a 2D segment of the velocity vector field around roughness elements in RMC3. The fluid impinges on the windward surface of the roughness elements and divides into different portions flowing around the element meeting behind it. This triggers flow perturbations which can generate small recirculation regions in the deep valley, as shown in Fig. 5(b), although shallow valleys are free of recirculation regions. The intensity of these recirculation regions reduces as the Reynolds number decreases. The observations of the present study showed the total absence of recirculation regions in RMC1 and RNC2 for the entire range of Reynolds number, with no recirculation regions in RMC 3 at *Re* < 800. Pelević and van der Meer [33] also observed no recirculation regions for low roughness and Reynolds numbers between $200\le Re\le 600$. Figure 5 also shows that the flow over roughness structure is streamlined, as expected in laminar flows; therefore, minimal flow mixing close to the rough surface is anticipated.

According to the literature [33, 70], 2D roughness models fail to accurately model the actual dynamics of rough-wall flows and roughness effects. This could be largely due to the existence of a 3D flow structure near the roughness, as seen in Fig. 5. The roughness structure forces the fluid to flow in a spanwise direction that cannot be captured in 2D models and false recirculation regions may emerge in the results instead.

**Fig. 6:**
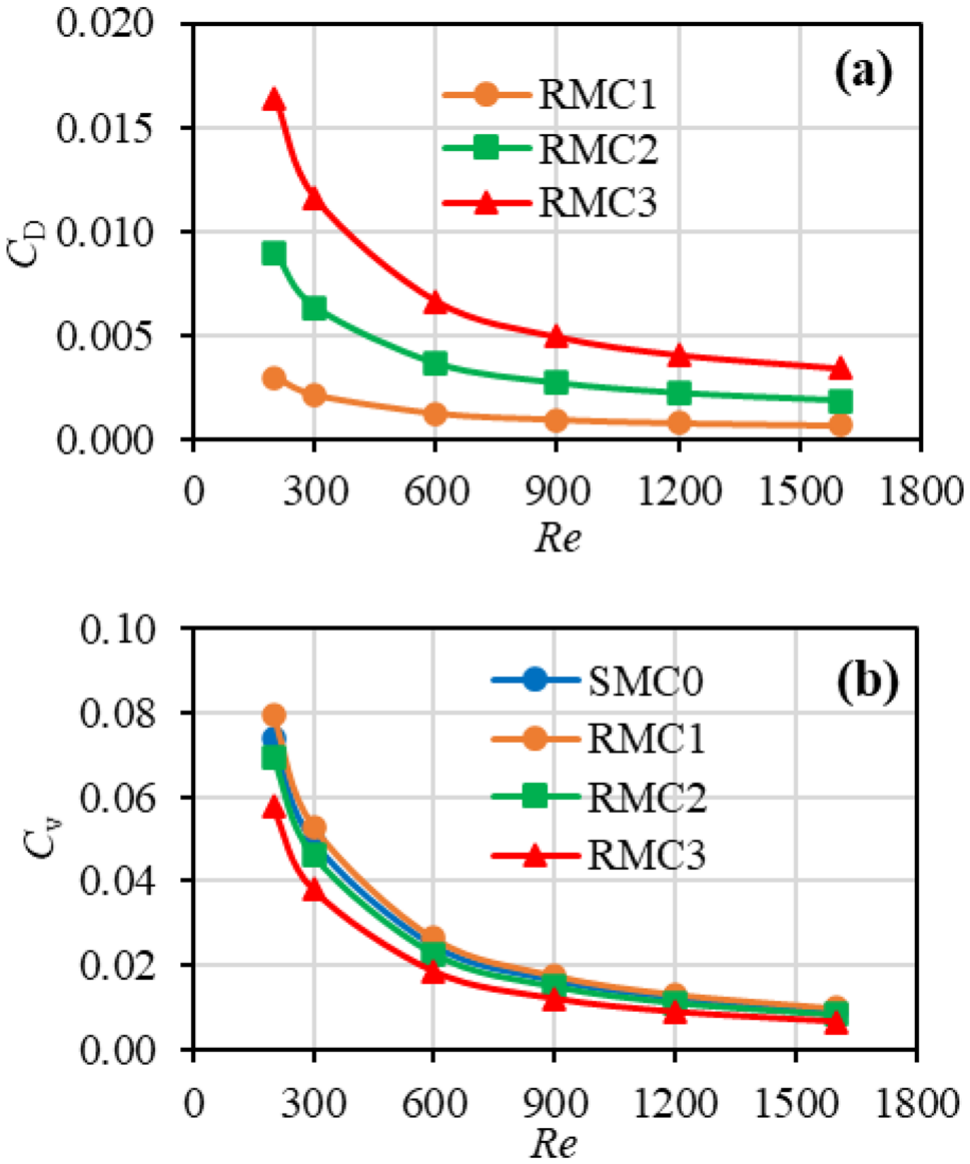
Variation of **a** form drag coefficient (${C}_{D}$) and **b** viscous drag coefficient (${C}_{v}$) with Re

**Fig. 7:**
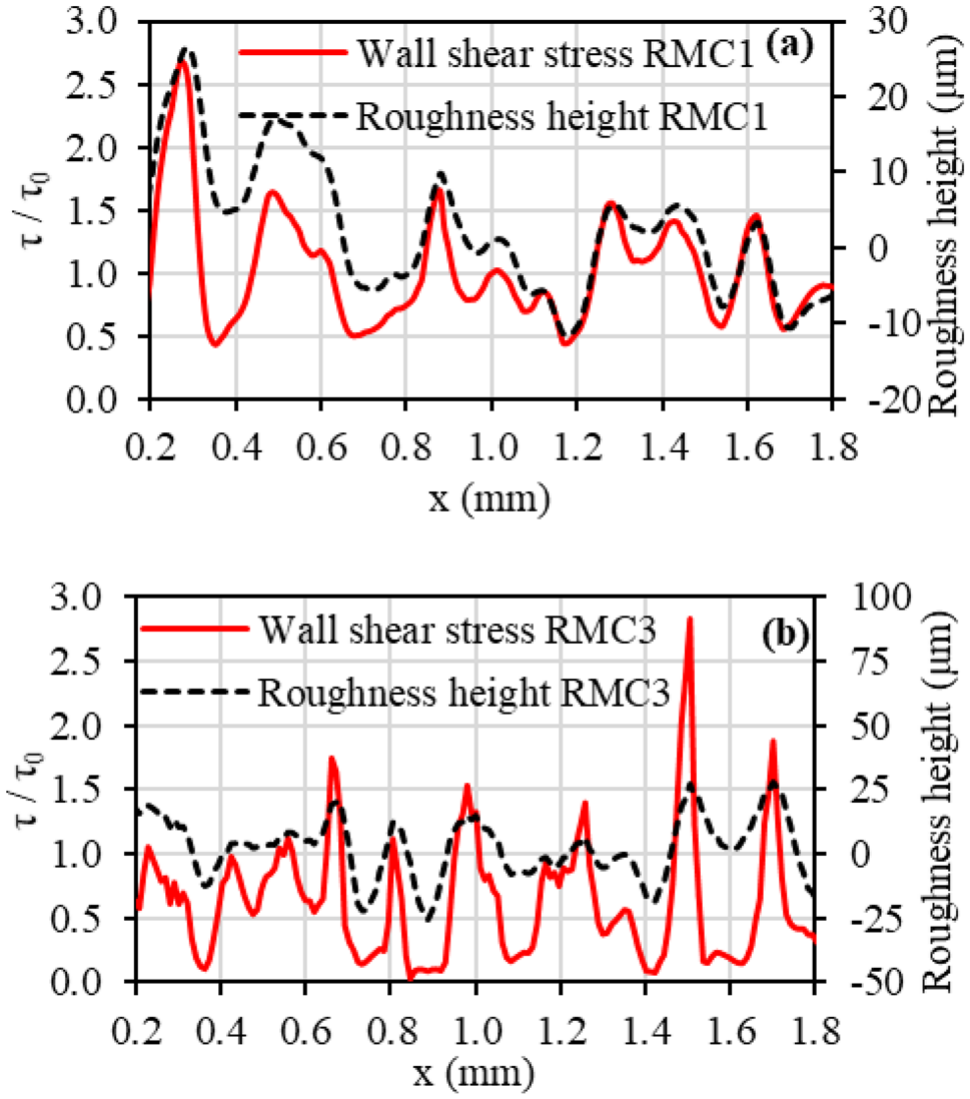
Variation of local viscous shear stress ratio with roughness profile in the streamwise direction at the centre of **a** RMC1 and **b** RMC3 from 0.2 to 1.8 mm at *Re* = 200

Roughness elements increase the friction drag on the flow via two mechanisms: (i) form drag as a result of the projection of the roughness elements into the stream, and (ii) viscous drag, as a result of the fluid viscous shear stress. Figure 6(a) indicates the variation of the form drag coefficient (${C}_{D}$) in the rough minichannels as a function of Reynolds number. The fluid impingement on roughness elements increases the form drag via increased windward face area. Since the form drag increases with roughness, it is negligible in RMC1 and reaches its highest value in RMC3. Figure 6(b) indicates the variation of viscous drag coefficient (${C}_{v}$) in the rough minichannels as a function of Reynolds number. Since the flow is laminar, the differences in viscous drag between channels are of low magnitude and the slight reduction in ${C}_{v}$ with roughness can be attributed to the reduced shear stress in the low-velocity flow regions in roughness valleys (See Fig. 7).

Comparing Fig. 6(a) and (b) reveals several findings: (i) since the variation of the viscous drag between channels is marginal, the increased flow friction in rough channels (observed in Fig. 4) is more likely due to the increase in form drag by roughness; (ii) the magnitude of the form drag is substantially lower than that of friction drag, meaning the friction is more sensitive to fluid viscosity than roughness; thus the increased flow friction by roughness is low, at a maximum of around 3% (as shown in Fig. 4).

Figure 7 illustrates the variation of local viscous shear stress ratio (at *Re* = 1600) with roughness structure in the streamwise direction *x* between 0.2 to 1.8 mm at the centre of the RMC1 (Fig. 7(a)) and RMC3 (Fig. 7(b)). The vertical axis at the left represents the shear stress ratio of the rough channel ($\tau$) and baseline smooth channel (${\tau }_{0}$), while the vertical axis at the right represents the variation of roughness heights. It can be seen that the viscous shear stress follows the roughness structure, increasing over roughness peaks and decreasing in roughness valleys. The low-velocity flow regions in roughness valleys reduce the shear stress, whereas high-velocity gradient flows over roughness peaks increase shear stress. Figure 7(a) shows an almost equal increase and decrease in the shear stress in RMC1, leading to a marginal increase in the viscous friction compared to SMC0 (See Fig. 6(b)). However, by increasing the roughness height, the reduction of viscous shear in roughness valleys outweighs any increase over the roughness peaks, as illustrated in Fig. 7(b). The majority of the shear stress graph in Fig. 7(b) is positioned below unity, providing average viscous friction lower than that of SMC0 that was observed in Fig. 6(b)).

**Fig. 8:**
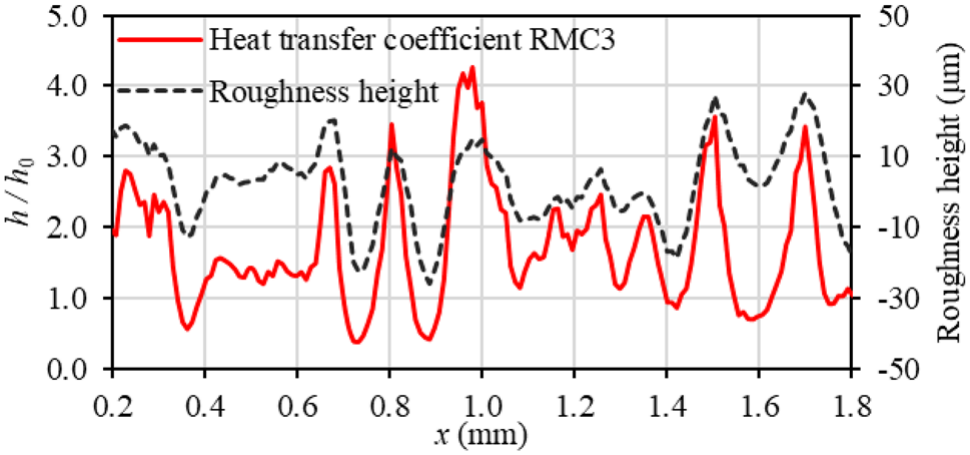
Variation of local heat transfer coefficient ratio with roughness profile in the streamwise direction at the centre of RMC3 from 0.2 to 1.8 mm at *Re* = 1600

Figure 8 illustrates the variation of heat transfer coefficient ratio (at *Re* = 1600) with roughness profile in the streamwise direction on the rough surface at *x* between 0.2 to 1.8 mm at the centre of the RMC3 channel. The vertical axis at the left represents the local heat transfer coefficient ratio of the RMC3 ($h$) and that of the baseline smooth channel (${h}_{0}$), while the vertical axis at the right represents the variation of roughness heights. It can be seen that heat transfer is enhanced over roughness peaks and reduced in roughness valleys. One reason for the enhanced heat transfer is the flow impingement over the windward face of roughness elements and flow acceleration over and between roughness peaks, as shown in Fig. 5(a). Another reason for heat transfer enhancement over roughness peaks is the higher energy transfer from the roughness elements to the channel wall. According to Pelević and van der Meer [33], the roughness elements possess higher thermal conductivity than the fluid which creates a thermal-flow shortcut to the bottom wall of the channel. The main reason for heat transfer reduction in roughness valleys is the presence of low-velocity flow in roughness valleys; however, the overall heat transfer is enhanced over roughness because the enhancement effects outweigh the reduction effects. With reference to the smooth channel case, the fluctuation of the local heat transfer coefficient is the summation of local heat transfer enhancements and local heat transfer reductions [33]. The heat transfer coefficient fluctuates around an average value greater than that of the smooth surface [71]. By increasing the roughness height, the number of peaks and their height increases, leading to higher heat transfer enhancement. These observations are in agreement with the findings of Pelević and van der Meer [33] and Lu et al. [42] for the heat transfer coefficient.

**Fig. 9:**
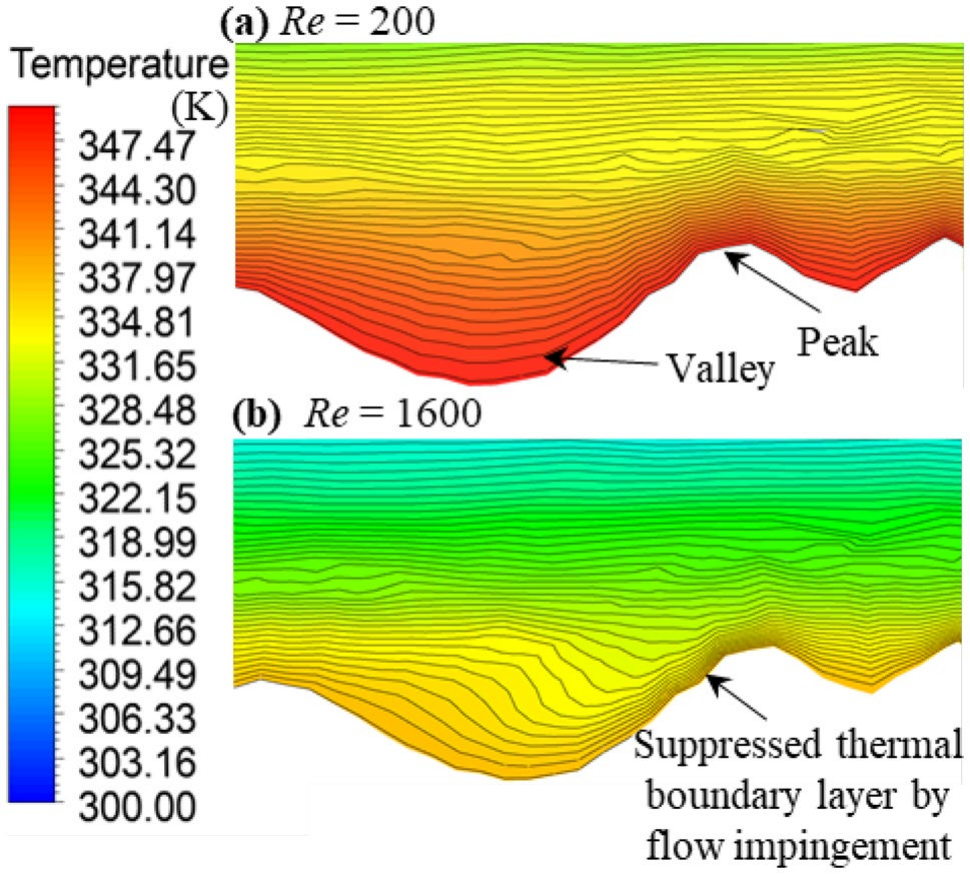
2D segments of temperature contour at **a** Re = 200 and **b** Re = 1600

Figure 9 illustrates 2D segments of temperature contours in RMC3 for the lowest and highest Reynolds numbers studied (i.e., *Re* = 200 and *Re* = 1600). At *Re* = 200, low-velocity flow regions prevail in the roughness valleys, leading to flow stratification in these regions and increasing the thermal boundary layer thickness as shown in Fig. 9(a). Due to the reduced fluid velocity at *Re* = 200, flow impingement on the windward face of roughness is insufficiently strong to disrupt flow stratification. Increasing the flow velocity can affect the flow impingement by increasing its intensity suppressing the growth of the thermal boundary layer, leading to higher temperature gradients close to the surface and hence enhancing heat transfer. At *Re* = 1600, flow impingement on the windward face brings the temperature contours closer to the surface, as shown in Fig. 9(b), reducing the thickness of the thermal boundary layer in the region and enhancing heat transfer.

Figure 9 demonstrates that, due to a higher velocity gradient over roughness peaks, the thermal boundary layer is suppressed in the region. Comparing the thermal boundary layer thickness in Fig. 9(a) and (b), shows that increasing the Reynolds number leads to the suppression of the thermal boundary layer over roughness peaks, which is another reason for heat transfer enhancement. A similar observation was reported by Pelević and van der Meer [33] and Lu et al. [42].

**Fig. 10:**
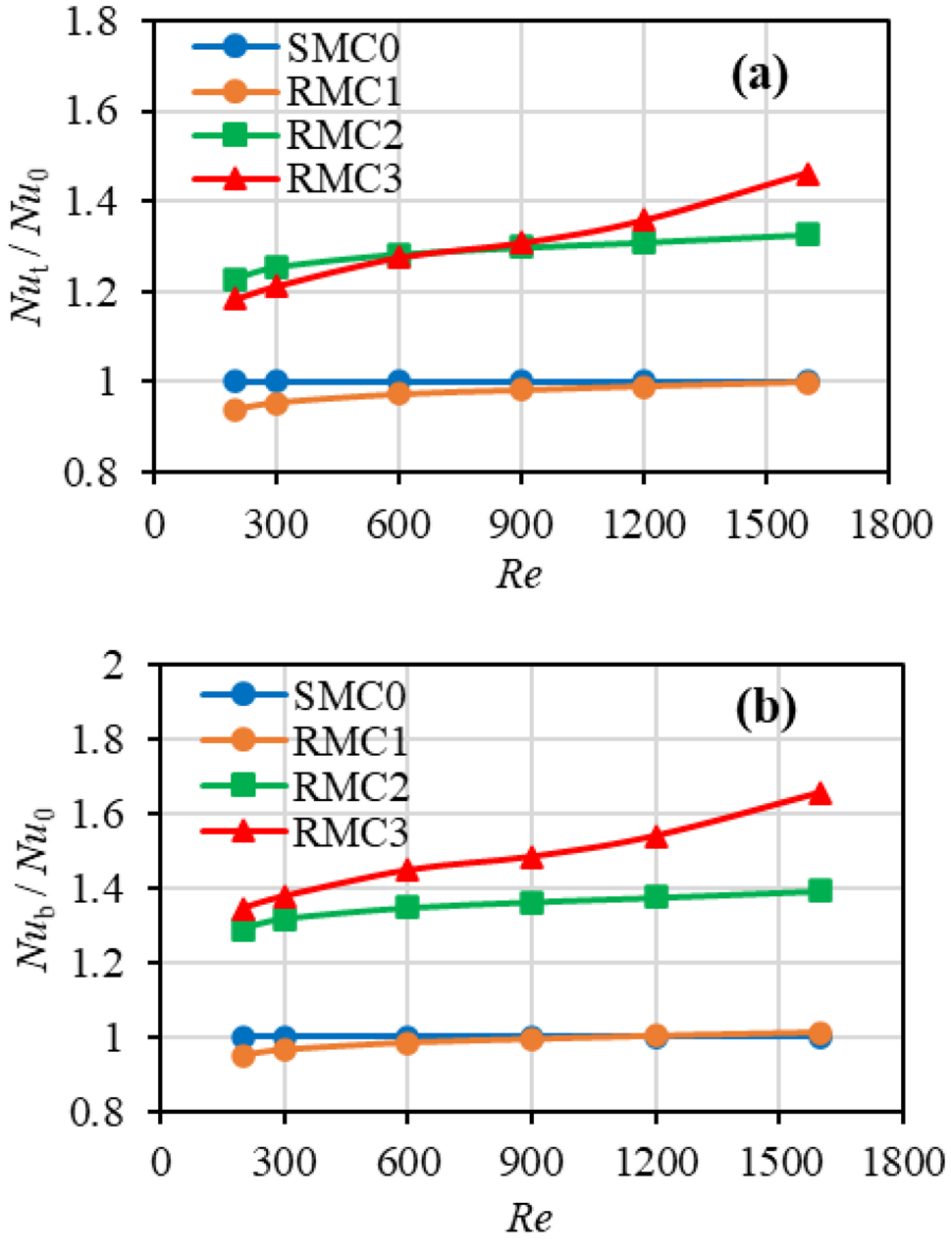
Impact of Reynolds number and roughness on the Nusselt number ratio with respect to **a** base area, and **b** total wetted area

Figure 10 shows the variation of the Nusselt number ratio as a function of Reynolds number ranging from 200 to 1600. Unlike the smooth channel (SMC0), where the Nusselt number is independent of the Reynolds number (conventional theory), in rough channels, the Nusselt number increases with the Reynolds number.

The vertical axis in Fig. 10(a) represents the Nusselt number ratio with respect to the total wetted surface area (${Nu}_{t}$). At *Re* = 200, the ${Nu}_{t}$ ratio of RMC1 is approximately 6% lower than that of the smooth (SMC0), but on increasing the Reynolds number to 1600, it returns to a value approaching that of SMC0. A similar reduction in heat transfer due to roughness was reported in the experimental studies of Shen et al. [51] and Qu et al. [61]. This reduction in ${Nu}_{t}$ suggests that at low Reynolds numbers, the heat transfer enhancement over roughness peaks does not exceed the decreases associated with the roughness valleys. Increasing the roughness height is seen to enhance the heat transfer; with the ${Nu}_{t}$ ratio of RMC2 of 1.23 at *Re* = 200 increasing somewhat slowly to 1.32 at *Re* = 1600. For RMC3, however, while initially lower at 1.19 at *Re* = 200 increases more sharply to a value of 1.46 at *Re* = 1600. The ${Nu}_{t}$ / ${Nu}_{0}$ ratios suggest that at lower *Re* values, with moderate roughness values can improve heat transfer, yet even low roughness is detrimental.

Figure 10(b), the ratio of the Nusselt number with respect to the base area (${Nu}_{b}$) is plotted against Reynolds number. At *Re* = 200, the ${Nu}_{b}$ of RMC1 is roughly 5% lower than that of the smooth SMC0, and with increasing Reynolds number, it also climbs to a value (1.24%) slightly higher than that of SMC0. The ${Nu}_{b}$ ratio of RMC2 and RMC3 are higher than that of RMC0 at *Re* = 200 (1.29 and 1.34, respectively), and as the Reynolds number increases, they also increase up to 1.39 and 1.65.

Comparing the Nusselt values in Fig. 10(a) and (b) indicates that there is a noticeable difference between the ${Nu}_{b}$ and ${Nu}_{t}$ values, mainly due to increased wetted area by roughness, showing that roughness can be a significant contributing factor in heat transfer enhancement. In both graphs, for low-level roughness of RMC1, little enhancement is shown above *Re* = 900, and below *Re* = 900, the heat transfer is essentially lower than that of the smooth channel. At moderate roughness levels of RMC2, the heat transfer is seen to improve, and a small positive influence of Reynolds Number is apparent. In the roughest channel (RMC3), both ${Nu}_{t}$ and ${Nu}_{b}$ enhancement show a greater positive influence with increasing Reynolds number. While both *Nu* plots for RMC3 and RMC2 have similar trends, at *Re* < 900, the ${Nu}_{t}$ of RMC3 drops below that of RMC2. This suggests that at lower Reynolds numbers (*Re* = 900), an optimal roughness level may exist for heat transfer enhancement.

**Fig. 11:**
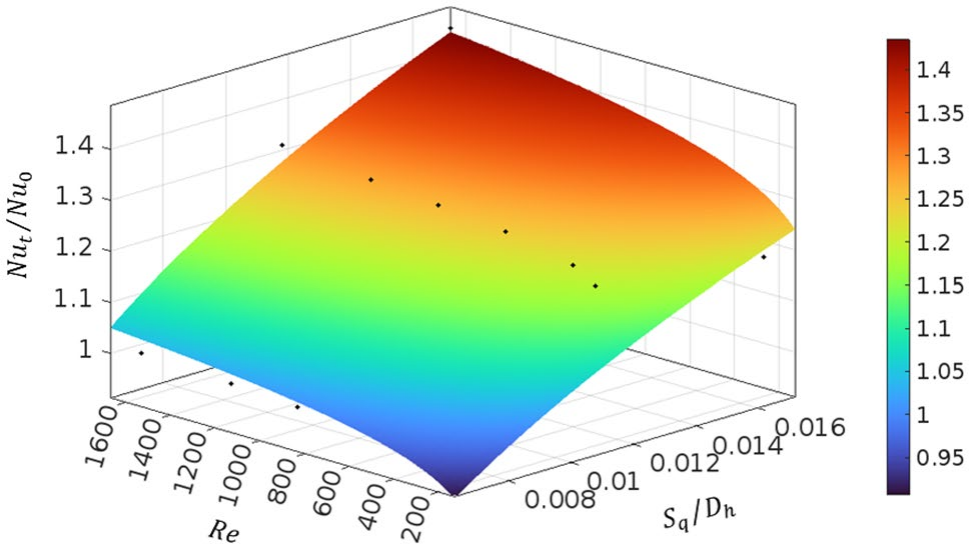
3D plot of the Nusselt number ratio versus Reynolds number and relative roughness (${S}_{q}/{D}_{h}$). Black points are the actual numerical data (some of the points are hidden below the curve) and the surface plot represents the fitted curve on the data

Figure 11 illustrates the 3D plot of the variations of the Nusselt number ratio by Reynolds number and relative roughness (${S}_{q}/{D}_{h}$). The black points are the actual numerical data (some of the points are hidden below the curve) and the surface plot represents the fitted curve on the data, which can be expressed by

18 $$\frac{{Nu}_{t} }{{Nu}_{0} }=3.331{ \left({S}_{q}/{D}_{h}\right)}^{0.3117}{Re}^{0.05723}$$

This correlation is valid for ${0.0068\le S}_{q}/{D}_{h}\le 0.0168$ and $200\le Re\le 1600$ within a root mean square error of 7.73%. Figure 11 shows that the Nusselt number is more sensitive to changes in ${S}_{q}/{D}_{h}$ rather than the Reynolds number.

**Fig. 12:**
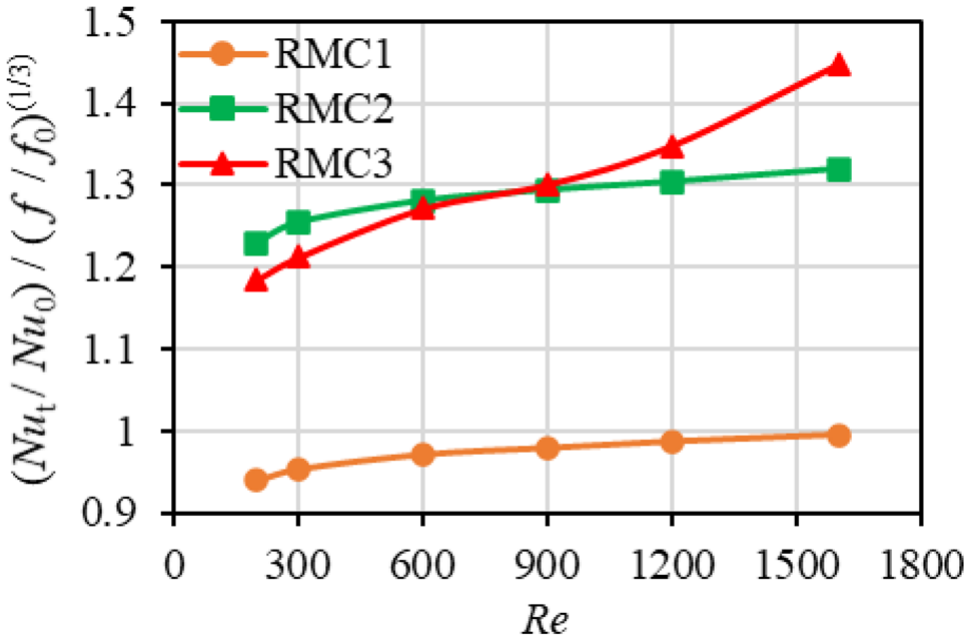
Variation of thermal performance factor with Reynolds number

The thermal performance factor of the channels is illustrated in Fig. 12. The thermal performance factors of RMC2 and RMC3 are higher than unity, meaning the heat transfer enhancement due to roughness outweighs the penalty of flow friction. However, it is less than unity for RMC1, showing its relatively poor thermal performance. At *Re* = 200, the thermal performance of RMC3 is 3.6% lower than that of RMC2, and with increasing the Reynolds number, it overtakes the RMC2 value at *Re* ≈ 800 and outperforms it by 9.6% at *Re* = 1600. Figure 12 also suggests that, at low Reynolds numbers, very low relative roughness (${S}_{q}/{D}_{h}=0.0065$) may worsen the thermal performance to a value lower than that of smooth surfaces. However, further increase in the roughness appears to show a general improvement in heat transfer, until increasing roughness beyond a certain parameter, again leads to a decrease in heat transfer. This emphasises the importance of roughness at a range of Reynolds number and relative roughness and the existence of optimum values.

## Conclusion

This paper performed a CFD study of laminar convective heat transfer in square minichannels with surface relative roughness $({S}_{q}/{D}_{h})$ of 0.0068, 0.0113, and 0.0167, and Reynolds numbers ranging from 200 to 1600. The impact of roughness and Reynolds number on the flow and heat transfer characteristics were investigated and the main conclusions are summarised as follows.

For low roughness and at low Reynolds numbers, simulations indicate that the heat transfer is lower than that of smooth channels and that at low Reynolds numbers, an optimal relative roughness value exists. At higher Reynolds, again low roughness provides none or a very marginal increase in heat transfer. Within the parameters studied, as Reynolds number and roughness both increase, heat transfer and flow friction were seen to increase. For relative roughness of 0.0068, 0.0113, 0.0167, the heat transfer was enhanced by 0.03%, 32.74%, and 46.05%, while the friction factor increased by 1.56, 1.71, and 2.91% respectively. The small friction factors at low roughness indicate the absence of complex flow patterns such as recirculation regions. For relative roughness of 0.0167 small recirculation regions in some of the deep valleys were observed at *Re* > 800, which, along with the higher flow drag induced by the roughness elements, is the reason for relatively larger flow friction in this channel. The flow friction is more sensitive to viscous shear force within the fluid stream than the form drag over the roughness elements.

The reason for heat transfer enhancement in the rough channels with increasing Reynolds number follows the suppression of the thermal boundary layer due to enhanced flow impingement and increased velocity gradient over roughness elements. The heat transfer enhancement by roughness can be attributed to several factors, including flow impingement against the roughness elements, as well as the improved thermal-flow path, and increased heat transfer area by roughness elements. However, further studies are required to investigate the impact and the degree of influence of each contributing factor.

The thermal performance analysis demonstrates the capacity of high roughness for heat transfer enhancement in minichannels, yet with only a marginal increase in flow friction. The findings of this study are useful in the additive manufacture of metallic rough minichannels with enhanced thermal performance for various applications such as conformal cooling of injection mould tooling and electronic cooling.

## Appendix A. Method of generating random roughness

The characterization of irregular roughness is complex; however, the morphology of roughness can be represented by statistical parameters of roughness [26, 27], which can be used to create a mathematical representation. The probability of a point on the surface that has a height equal to $z$ is estimated by the Probability Density Function (PDF), given as [77]:

19 $${\int }_{-\infty }^{+\infty }P(z)dz=1$$

The central $\vartheta$^th^ moment of the PDF with zero-mean, ${m}_{\vartheta }$, provides the special characteristics of roughness, defined as [77]

20 $${m}_{\vartheta }={\int }_{-\infty }^{+\infty }{z}^{\vartheta }P(z)dz\ \mathrm{or\ in\ discrete\ form}\ {m}_{\vartheta }=\frac{1}{n}\sum_{i=1}^{n}{z}_{i}^{\vartheta }$$

The first four moments are equivalent representations of arithmetic-averaged height ${S}_{a}$, root-mean-square height ${S}_{q}$, skewness ${S}_{sk}$, and kurtosis ${S}_{ku}$ of roughness, respectively, defined as.

21 $$\begin{array}{cccc}S_a=m_1;&S_q=\sqrt{m_2};&S_{sk}=m_3/m_2^{3/2};&S_{ku}=m_4/m_2^4\end{array}$$

In order to generate surfaces with random roughness, a random heightmap $z(x,y)$ of the surface profile at the coordinates $x$ and $y$ must be generated based on a specified profile. The spectral characteristics of the roughness profile can be evaluated by the Autocorrelation Function (ACF). The ACF of a surface with a profile $z(x,y)$ is defined as a convolution of the surface with itself, shifted by (${\beta }_{x},{\beta }_{z}$) [77]

22 $$R\left({\beta }_{x},{\beta }_{y}\right)=\underset{\begin{array}{c}{L}_{x}\to \infty \\ {L}_{y}\to \infty \end{array}}{\mathrm{lim}}\left[\frac{1}{{L}_{x}{L}_{y}}{\int }_{0}^{{L}_{x}}{\int }_{0}^{{L}_{y}}z\left(x,y\right)z(x+{\beta }_{x},y+{\beta }_{y})dxdy\right],$$

where ${L}_{x}$ and ${L}_{y}$ are the sampling length and ${\beta }_{x}$ and ${\beta }_{y}$ are the Autocorrelation Lengths (ACL). $R\left(\mathrm{0,0}\right)={{S}_{q}}^{2}$ is the maximum value, and as ACL tends to infinity, $R\left({\beta }_{x},{\beta }_{y}\right)$ asymptotically decays to zero. In practice, the ACL is defined as the horizontal distances in $x$ and $y$ directions in which the autocorrelation function reduces to 10% of its value [78]. The ACF of a rough surface is approximately exponential for a large variety of engineering surfaces [78], and can be approximated by the Gaussian distribution, expressed by

23 $$R\left(x,y\right)=\mathrm{exp}\left[-2\left[{\left(\frac{x}{{\beta }_{x}}\right)}^{2}+{\left(\frac{y}{{\beta }_{y}}\right)}^{2}\right]\right]$$

The Gaussian profile is associated with a heightmap with skewness ${S}_{sk}=0$, and kurtosis ${S}_{ku}=3$. The skewness indicates the departure of roughness elements from symmetry, while kurtosis represents the sharpness of the roughness profile. Therefore, Gaussian roughness has symmetric profiles with equally distributed peaks and valleys, and also ${S}_{q}\approx {S}_{a}\sqrt{\pi /2}$ [71, 73]. The Fourier transform of the ACF provides the Power Spectral Density (PDS) of roughness, which plays a key role to define a process for roughness generation [33, 42, 48].

A convenient way to generate surfaces with given statistical parameters would be to create surfaces with predetermined ACF. The ACF can reflect the surface profile height and the effective wavelength while differentiating the periodicity and randomness of the profile to hold the shape characteristics of the profile. Therefore, the ACF can describe the entire surface shape characteristics. According to Patir [79], an (N,M) matrix of rough amplitudes that follows a specified statistical distribution and a given (n,m) ACF can be generated using an (N + n, M + m) matrix of independent identically distributed random numbers with mean value equal to zero and unit standard deviation (root-mean-square) according to the following transformation:

24 $$\begin{array}{c}z\left(\mathrm I,\mathrm J\right)=\sum_{k=1}^n\sum_{l=1}^m\theta\left(\mathrm k,\mathrm l\right)\eta\left(\mathrm I-\mathrm k,\mathrm J-\mathrm l\right);\\\begin{array}{cccc}\mathrm I=1,2,\dots,N;&\mathrm J=1,2,\dots,M;&k=1,2,\dots,n;&l=1,2,\dots,m;\end{array}\end{array}$$

where $\theta (\mathrm{I},\mathrm{J})$ are coefficients to be determined to produce the desired ACF and $\eta$ is an input matrix of independent random numbers and has unit standard deviation; therefore, the ACF is defined as [78, 79]

25 $$\begin{aligned}R\left(p,q\right)& =\frac{1}{NM}\sum\limits_{k=1}^{N-p}\sum\limits_{l=1}^{M-q}\theta \left(\mathrm{k},\mathrm{l}\right)\theta \left(k+p,l+q\right);\ \\ p & = \mathrm{0,1},2, \dots , n-1;\ q= \mathrm{0,1},2, \dots , m-1;\end{aligned}$$

Eq. (25) is a nonlinear system of equations with $n\times m$ dimensions. This paper developed a filtering method based on the Fast Fourier Transform (FFT), implemented in MATLAB, to solve Eq. (25). According to the definition, the PSD of the height map can also be obtained by applying the Fourier transform of the ACF (Eq. (25)). The relation between the PSD of the output matrix, Z, and the PSD of the input matrix, H, can be obtained by applying the Fourier transform to Eq. (24), given by

26 $$Z\left({\omega }_{x},{\omega }_{y}\right)=\Theta \left({\omega }_{x},{\omega }_{y}\right)\mathrm{\rm H}\left({\omega }_{x},{\omega }_{y}\right);$$

where $\Theta$ is the Fourier transform of $\theta ,$ which is referred as to the transfer function of the system, given by

27 $$\begin{aligned}\Theta \left({\omega }_{x},{\omega }_{y}\right)& =\sum\limits_{k=1}^{n}\sum\limits_{l=1}^{m}\theta \left(\mathrm{k},\mathrm{l}\right)\mathrm{exp}\left(-ik{\omega }_{x}\right)\mathrm{exp}\left(-ik{\omega }_{y}\right);\ \\ {\omega }_{x} & = \mathrm{1,2}, \dots ,n;\ {\omega }_{y}= \mathrm{1,2}, \dots , m\end{aligned}$$

where $$i=\sqrt{-1}$$.

Since $\eta$ is a matrix of independent random numbers, its PSD is a constant, i.e. $\mathrm{H}\left({\omega }_{x},{\omega }_{y}\right)=c$; hence

28 $$\Theta \left({\omega }_{x},{\omega }_{y}\right)=Z\left({\omega }_{x},{\omega }_{y}\right)/c$$

The initial height matrix with a gaussian distribution can be obtained by applying the inverse Fourier transform to Eq. (26)

29 $${z}_{0}\left(\mathrm{I, J}\right) =\frac{1}{nm}\sum\limits_{{\omega }_{x}=1}^{n}\sum\limits_{{\omega }_{y}=1}^{m}\left[\Theta \left({\omega }_{x},{\omega }_{y}\right)\mathrm{\rm H}\left({\omega }_{x},{\omega }_{y}\right)\right]\mathrm{exp}\left(-ik{\omega }_{x}\right)\mathrm{exp}\left(-ik{\omega }_{y}\right)$$

A random matrix of height (heightmap) with specified ACF can be generated using the following algorithm:

1. specify a 2D random matrix $\eta \left(\mathrm{I},\mathrm{J}\right)$ (with zero mean and unite standard deviation);
2. applying FFT to $\eta \left(\mathrm{I},\mathrm{J}\right)$ to find the matrix $\mathrm{\rm H}$;
3. discretise the corresponding Gaussian ACF (Eq. (23));
4. determine the PSD of the output matrix, $Z,$ by applying FFF to the discretised ACF;
5. calculate the transfer function, $\Theta ,$ using Eq. (28) with $c=1;$
6. compute the initial height matrix, ${z}_{0}\left(\mathrm{I},\mathrm{J}\right)$, using Eq. (29).

The algorithm for generating Gaussian heightmaps can be summarised in the following equation

30 $${z}_{0} =\mathrm{IFFT}\left[\mathrm{FFT}\left(\eta \right).\mathrm{FFT}\left(R\right)\right]$$

where $\mathrm{FFT}$ and $\mathrm{IFFT}$ represent the fast Fourier transform and inverse fast Fourier transform, respectively. This initial height matrix has a root-mean-square of ${{S}_{q}}_{0}$ that may not be equal to desired ${S}_{q}$; therefore, the final height matrix (*z*) is obtained by normalising the initial matrix as $z=({S}_{q}/{{S}_{q}}_{0} ){z}_{0}$.

## Nomenclature

*A*: Base surface area
${A}_{c}$: Cross-sectional area
${A}_{t}$: Total wetted surface
${A}_{s}$: Roughness surface area
${C}_{D}$: Form drag coefficient
${c}_{p}$: Specific heat capacity
${C}_{\nu }$: Viscous drag coefficient
$${D}_{h}$$: Hydraulic diameter
$f$: Darcy friction factor
$${F}_{D}$$: Drag force
$${F}_{\nu }$$: Shear force
$h$: Local convective heat transfer coefficient
$$\mathrm{H}$$: PSD of $$\eta$$
$$k$$: Thermal conductivity
$$\dot{m}$$: Mass flowrate
${m}_{\vartheta }$: The central $\vartheta$ ^th^ moment of the PDF
${Nu}_{b}$: Average Nusselt number with respect to the base area
${Nu}_{t}$: Average Nusselt number with respect to the wetted surface area
$$p$$: Pressure
$P$: Probability Density Function (PDF)
$$\mathrm{PF}$$: Performance factor
$$Pr$$: Prandtl number
$q$: Local heat flux
$Q$: Total heating power
$R$: Autocorrelation function (ACF)
$$Re$$: Reynolds number
${S}_{a}$: Arithmetic-averaged height
${S}_{q}$: Root-mean-square height
${S}_{ku}$: Kurtosis of roughness
${S}_{sk}$: Skewness of roughness
$S$: Power Spectral Density (PDS)
$$T$$: Temperature
$${T}_{in}$$: Inlet temperature
${T}_{\mathrm{f}}$: Volume-averaged fluid temperature
$${T}_{w}$$: Wall temperature
$$U$$: Average velocity
$\overrightarrow{V}$: Velocity vector field
$$x$$: Streamwise coordinate
$$y$$: Spanwise coordinate
$$z$$: Heightmap matrix
$$Z$$: PSD of $$z$$

## Greek letters

${\beta }_{x}$: Streamwise autocorrelation Length
${\beta }_{y}$: Spanwise autocorrelation Length
$$\rho$$: Density
$\tau$: Wall shear stress
$$\eta$$: Random matrix
$\theta$: Matrix of coefficients
$$\Theta$$: Transfer function
$\mu$: Dynamic molecular viscosity
$$\forall$$: Volume

## Abbreviations

ACF: Autocorrelation Function
ACL: Autocorrelation Length
AM: Additive Manufacturing
CFD: Computational Fluid Dynamics
FVM: Finite Volume Method
FFT: Fast Fourier Transform
IFFT: Inverse Fast Fourier Transform
NRMSE: Normalised Root Mean Square Error
PBF: Powder Bed Fusion
PDF: Probability Density Function
PDS: Power Spectral Density
RMS: Rough Minichannel
SMC: Smooth Minichannel
STL: Standard Tessellation Language
